# Impact of vestibular rehabilitation therapy on quality of life and cognitive function in individuals with chronic dizziness or vertigo

**DOI:** 10.1007/s00405-025-09382-0

**Published:** 2025-04-07

**Authors:** Anupriya Ebenezer, Kaushlendra Kumar, Mohan Kumar Kalaiah, Deviprasad Dosemane, M. Ramiz Malik

**Affiliations:** 1 Department of Audiology and Speech Language Pathology, Kasturba Medical College Mangalore, Manipal Academy of Higher Education, Karnataka, Manipal, 576 104 India; 2 Department of Otorhinolaryngology, Kasturba Medical College Mangalore, Manipal Academy of Higher Education, Karnataka, Manipal, 576 104 India

**Keywords:** Dizziness, Vertigo, Vestibular rehabilitation therapy, P300, Dizziness handicap inventory, Digit span test, Task switching test

## Abstract

**Purpose:**

Dizziness and vertigo are among the most frequently reported complaints among patients across various medical and healthcare specialties. Studies have revealed that they are associated with cognitive impairments, particularly in older adults. While vestibular rehabilitation therapy (VRT) alleviates physical symptoms, its effects on cognitive function remain underexplored. Hence, this study aims to assess the impact of VRT on the quality of life and cognitive performance of individuals with chronic dizziness or vertigo.

**Methods:**

This was a randomized control trial in which 60 participants experienced chronic dizziness or vertigo. The participants were assigned to either the medication-only group receiving betahistine or the VRT + medication group receiving VRT combined with betahistine. Quality of life was measured via the Dizziness Handicap Inventory (DHI). Cognitive performance was assessed via a digit span test, task-switching test, and recording of P300 response.

**Results:**

The VRT + Medication group showed significant improvements in cognitive performance, particularly in the digit span and task-switching tests, with reduced P300 response latency and increased amplitude. No significant cognitive changes were observed in the medication-only group. Both groups showed improvement in quality of life, with a greater reduction in DHI scores observed in the VRT + Medication group.

**Conclusion:**

VRT combined with medication significantly improves cognitive function and quality of life in individuals with chronic dizziness or vertigo. These findings suggest that VRT not only addresses physical symptoms but also enhances cognitive performance, highlighting its potential as a comprehensive therapeutic approach.

**Trial registration:**

The study protocol was registered in the Clinical Trial Registry of India (CTRI number: CTRI/2020/03/023934).

## Introduction

Dizziness and vertigo are among the most common complaints reported by patients seeking care from multiple medical and healthcare specialties [1]. While dizziness has a lifetime prevalence of 17–30%, vertigo is reported in 3–10% of the population. With the increasing prevalence among elderly individuals and the aging of the general population, a further increase in vertigo cases is anticipated [2]. Previous studies reported that a large proportion (approximately 20–50%) of dizziness and vertigo is caused by peripheral vestibular diseases such as BPPV, vestibular neuritis, and labyrinthitis. Other causes include cardiovascular diseases, systemic infections, psychiatric conditions, metabolic disturbances, and medications [3].

Recently, significant attention has been directed toward the relationship between vestibular dysfunction and cognitive processes. Research has explored this area through various streams. One focuses on the prevalence of vestibular dysfunction in populations with cognitive function deficits such as dementia or Alzheimer’s disease [4, 5]. Another line of research has investigated the cognitive abilities of individuals with vestibular disorders [6]. The “National Health and Nutrition Examination Survey” (2002) in the United States reported that vestibular dysfunction contributes to cognitive decline, significantly impacting daily living activities in older individuals and that vestibular dysfunction can impair cognitive functions, with affected individuals facing challenges in tasks related to visuospatial orientation, attention, memory, and executive function [7]. Substantial evidence from animal and human research has shown that vestibular dysfunction can impair cognitive functions, with affected individuals facing challenges in tasks related to visuospatial orientation, attention, memory, and executive function [8–12]. Studies highlight that vestibular dysfunction demands additional cognitive capacity to maintain balance, leading to increased competition for limited cognitive resources and reducing the ability to perform other tasks effectively. Kahneman’s capacity model of attention [13] supports this notion, emphasizing the finite nature of cognitive resources. Furthermore, studies have revealed that vestibular dysfunction may exacerbate depressive symptoms due to associated memory and concentration impairments [14]. Pavlou et al. emphasized the importance of assessing cognitive and functional gait performance in individuals with vestibular dysfunction. Their findings indicate reduced scores in certain cognitive domains during single- and dual-task functional gait assessments, particularly those involving numeracy and literacy tasks [15].

The P300 response is a human event-related potential (ERP) commonly used to assess cognitive processing. It is evoked via the oddball paradigm, where individuals respond to infrequent target stimuli amidst frequent non-targets. The P300 response wave reflects attentional and memory processes, indicating the brain’s cognitive response to stimulus evaluation and decision-making [16–18]. Recent studies have explored the utility of the P300 response as an objective method to assess the cognitive process among individuals with vestibular dysfunction. They found that cognitive impairment in vestibular dysfunction is linked to prolonged latency, reduced amplitude, or the absence of P300 response [17, 18].

Most of the time, in case of acute vestibular dysfunction, the spontaneous restoration mechanism of the vestibular function gets activated to achieve central compensation. However, it is not the same in all cases, where other management options, such as pharmacological treatment, vestibular exercise, physical therapy, surgery, or psychotherapy, are helpful in relieving vertigo or dizziness. Vestibular rehabilitation therapy (VRT) is an exercise-based approach designed to enhance gaze stability, postural stability, and sensory integration. It effectively improves balance in individuals with peripheral vestibular dysfunction, vestibular hypofunction, and those who are affected by central nervous system damage, including those with conditions such as Parkinson’s disease, multiple sclerosis, concussion, and cerebral palsy [19].

Additionally, cognitive behavioral therapy (CBT) has been shown to be effective in improving quality of life among people with dizziness [20, 21]. Edelman et al. (2012) reported a significant reduction in Dizziness Handicap Inventory (DHI) scores in individuals undergoing CBT for chronic subjective dizziness. However, no notable changes were observed in the psychological assessment outcomes [21]. Soberg et al. (2021) reported improvements in health-related quality of life following VRT for individuals with brain injuries, particularly those with balance issues and cognitive decline. Notably, subjective cognitive function scores improved post vestibular rehabilitation therapy in individuals with moderate traumatic brain injuries and vestibular dysfunction [22].

Despite these advancements in research related to cognitive function and dizziness or vertigo, there is a dearth of studies assessing the effect of vestibular rehabilitation therapy (VRT) on cognitive performance in dizziness- or vertigo-affected individuals. Given the well-established link between vestibular dysfunction and cognitive impairments, such as difficulties in visuospatial orientation, attention, memory, and executive function, understanding how VRT influences these cognitive domains is essential. This study aims to fill this gap by comparing cognitive function before and after VRT to evaluate its impact on cognitive performance. Furthermore, it seeks to investigate the correlation between quality of life, measured through Dizziness Handicap Inventory (DHI) scores, and cognitive performance. This research aims to provide valuable insights into how VRT not only alleviates physical symptoms but also enhances cognitive well-being, ultimately improving the overall quality of life of individuals with dizziness and vertigo.

## Method

The current study was carried out at the Department of Audiology and Speech Language Pathology, Kasturba Medical College, Mangalore, India, after ethical clearance from the Institutional Ethics Board. The study protocol was also registered in the Clinical Trial Registry of India. This study employed a randomized control trial design using an unstratified block randomization method with 21 permutations and 4 blocks, which was carried out via MS Excel. The randomization process included allocation concealment and blinding to ensure unbiased group assignment. The study recruited individuals who had experienced chronic dizziness or vertigo for at least one month. All participants demonstrated normal hearing sensitivity (< 25 dBHL) across specified frequencies for air and bone conduction, with no middle ear dysfunction, as confirmed by a Type ‘A’ tympanogram bilaterally.

A comprehensive evaluation by otolaryngologists, neurologists, and audiologists ruled out acute vestibular syndromes due to vestibular neuritis or strokes, other common vestibular pathologies such as Meniere’s disease, benign paroxysmal positional vertigo (BPPV), and superior semicircular canal dehiscence (SSCD). Vestibular evoked myogenic potentials and oculomotor function evaluation via videonystagmography (VNG) were carried out during this phase. Additionally, participants with any systemic illnesses such as diabetes mellitus, autoimmune diseases, or neurological conditions such as myasthenia gravis or multiple sclerosis were excluded. The subjects with overt cognitive deficits were screened using Mini-Mental State Examination (MMSE) and excluded, as such deficits may indicate more obvious cognitive disorders.

Although this profile of participants may resemble persistent postural-perceptual dizziness (PPPD), we refrained from using the term PPPD, as the selection criteria for participants did not fully meet the diagnostic standards established by the Bárány Society. Therefore, we refer to this condition as ‘chronic dizziness or vertigo’ in this context.

Participants were randomly allocated into two groups on the basis of the block randomization method. The first group, group 1, is the control group, which is named the medication-only group and does not receive any vestibular rehabilitation but receives medical intervention, i.e., betahistine (Tab. VERTIN), which is prescribed by an otolaryngologist and is based on body mass index and symptoms. On follow-up visits, the dosage was tapered down on review of symptoms. The second group was the experimental group of the study, named the VRT + Medication group, and its participants underwent a customized VRT program along with the prescribed medication, i.e., betahistine (Tab. VERTIN). The details of the VRT program are provided in the following section. All the participants in the study underwent assessments of quality of life and cognitive function before and after the intervention.

### Preintervention assessment

The preintervention phase involved assessing the impact of dizziness on quality of life using Dizziness Handicap Inventory (DHI). The cognitive assessments included a digit span test and a task-switching test, which were conducted via MATLAB, and the P300 response was recorded using continuous acquisition module (CAM) in intelligent hearing system.

The digit span test is used to measure working memory by presenting sequences of numerical digits, requiring participants to recall them in reverse order. The sequence length increased with correct responses and decreased with incorrect responses. The longest span of digits recalled correctly was considered the outcome measure of this test. The task switching test is used to evaluate cognitive flexibility and requires participants to respond to congruent or incongruent conditions on the basis of the direction of an arrow in an imaginary quadrant. Instructions were given to press ‘M’ for right-pointing arrows and ‘B’ for left-pointing arrows. The reaction time (in seconds) and the accuracy (in percentage) of the response were taken as outcome measures of the task switching test. Cognitive function was objectively assessed via P300 response, which was recorded via an autogenerated oddball paradigm with auditory stimuli. The participants were asked to focus on infrequent tones and discriminate them from frequently presented tones, with stimuli delivered monaurally to the right ear. EEG recordings were analyzed and interpreted by two experienced audiologists, and the latency and amplitude of the P300 response were considered outcome measures of this test.

### Vestibular rehabilitation therapy

Each participant in the VRT + Medication group received a home therapy plan, which was reviewed weekly, with follow-up sessions assessing symptoms via the “Effect of Vestibular Rehabilitation Therapy Questionnaire (EVRT-Q)” [23]. Based on symptom improvement, new exercises were provided, focusing on gaze stabilization and postural stability. Exercises were initially slow and progressively intensified based on the basis of the participant’s ability. The participants practiced VRT at home three times daily, accompanied by a bystander. Symptom-specific VRT strategies, including habituation exercises for dizziness, gaze stabilization for blurred vision, substitution and somatosensory training for imbalance, along with a hybrid approach integrating adaptation, habituation, and substitution, were applied. Exercise variables such as speed, direction, surface, and activity type were adjusted on the basis of prognosis. A 10-point Likert scale was used to monitor satisfaction and readiness for progression, with symptoms tracked to assess improvement.

### Postintervention assessment

Postintervention, both groups underwent DHI assessment to reassess the impact on their quality of life. The cognitive assessments (digit span test, task switching test, and P300 response) were re-administered to evaluate any changes in cognitive function following the intervention.

## Results

**Fig. 1:**
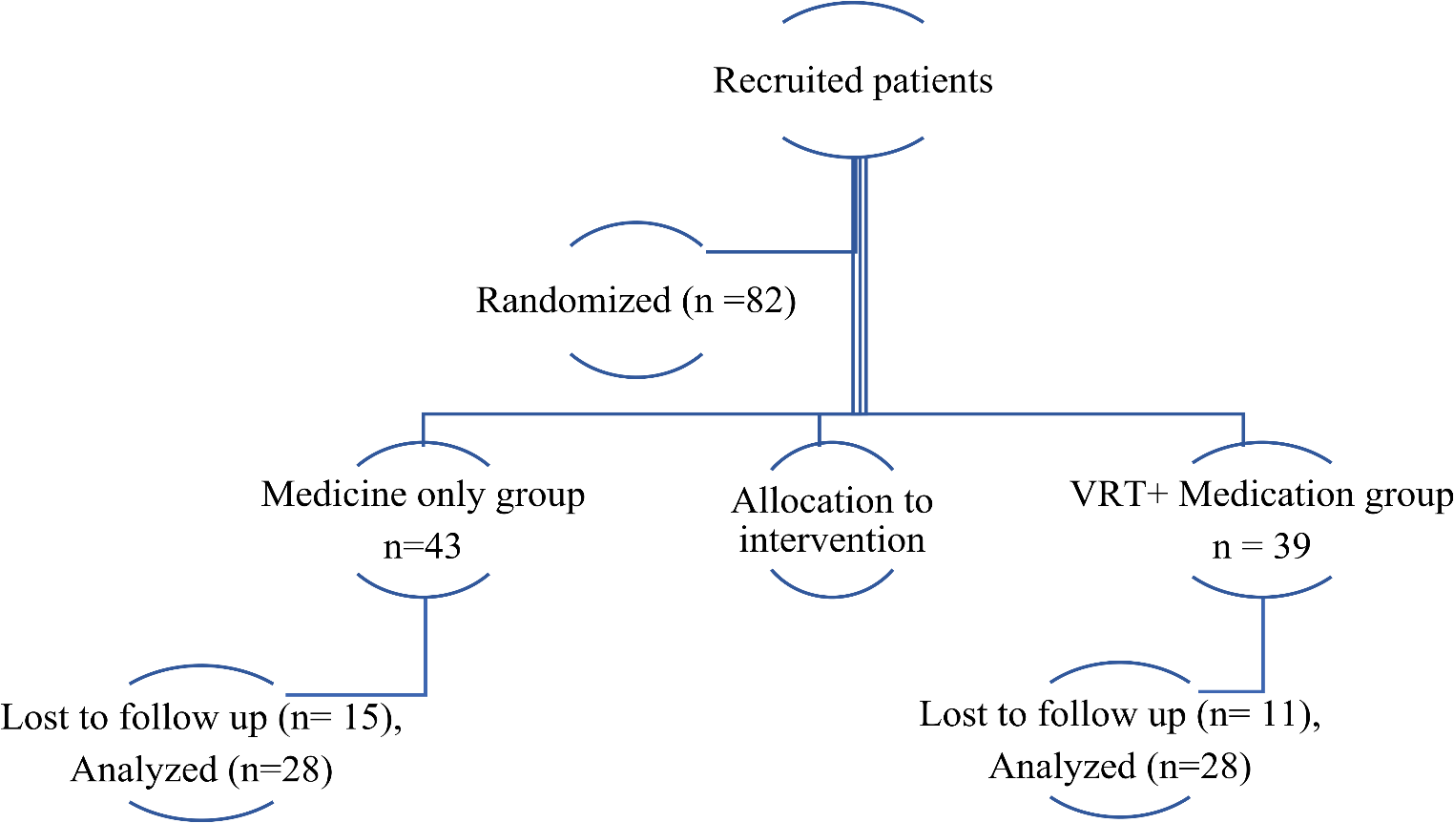
Flowchart showing the participant selection and randomization process

A total of 82 subjects were initially recruited for the study. However, the final analysis included 56 subjects, as some were lost to follow-up during data collection. Figure 1 depicts the process of final selection of participants.

**Fig. 2:**
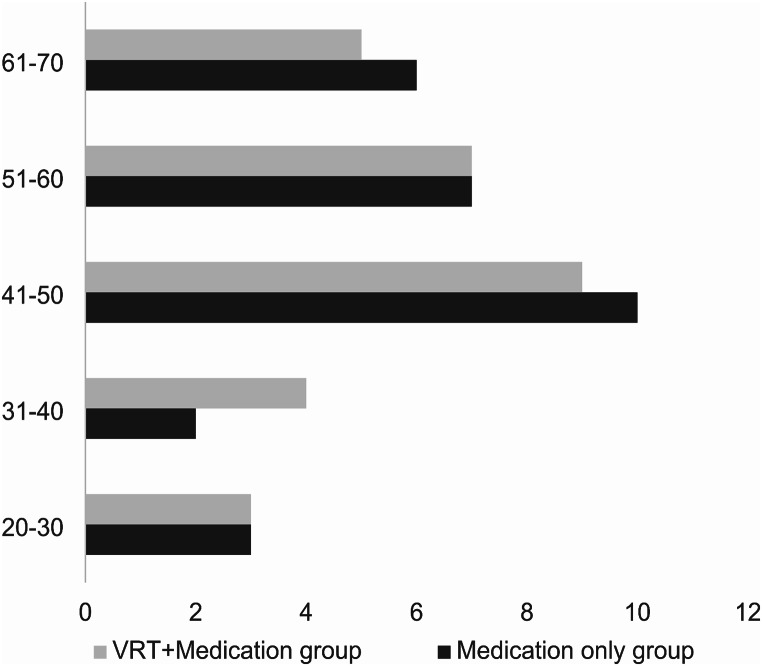
The age-wise categorization of participants

The mean age of the subjects in the medication-only group was 50.67 years (SD = 12.88), and in the VRT + Medication group was 46.81 years (SD = 12.13). The age wise categorization of participants is given in Fig. 2.

The core statistical analysis was conducted in two steps after the normality test. First, DHI and cognitive test scores or P300 response parameters were compared between the groups before and after the intervention. Later, comparison of DHI and cognitive test scores or P300 response parameters within each group to evaluate changes before and after the intervention.

### Effect of VRT on quality of life

The Shapiro‒Wilk test of normality indicated that the data were not normally distributed for some datasets. Therefore, to maintain consistency, nonparametric tests were used for all analyses.

The Mann‒Whitney U test results indicated no significant difference in DHI scores between the groups prior to the intervention (*U* = 381.50, *p* = 0.863). However, a significant difference was observed postintervention (*U* = 249, *p* = 0.019). Postintervention, the median DHI score for the VRT + Medication group decreased to 18 (IQR = 10–24) from 32 (18.50–50), whereas the median scores for the medication-only group were 31 (20–41.50) and 29 (IQR = 20–36) before and after the intervention, respectively.

A Wilcoxon signed-rank test was carried out to compare the preintervention DHI scores and postintervention scores. There was a significant reduction in DHI scores for the VRT + Medication group (Z=-4.174, *p* ≤ 0.05), with a large effect size (*r* ≈ 0.79), whereas the medication-only group showed no statistically significant difference (Z = -1.921, *p* = 0.055).

### Digit span test

Following the normality test, performance on the digit span test (DST) was compared between groups before and after the intervention. The Mann‒Whitney U test indicated no significant difference between the groups before the intervention (U = 365.50, *p* = 0.645). However, after the intervention, a significant difference emerged between the groups (U = 222.50, *p* = 0.003). Within-group comparisons using the Wilcoxon signed-rank test revealed a significant improvement in DST performance in the VRT + Medication group (Z = -3.987, *p* < 0.05), whereas no significant change was observed in the Medication-only group (Z = -1.414, *p* = 0.157).

### Task-switching test

The task-switching test included two outcome measures (reaction time and accuracy) evaluated under congruent and incongruent conditions. As the normality test revealed that some data sets were not normally distributed, non-parametric tests were employed for comparisons.

For the congruent condition, no significant between-group differences were observed before (U = 288.50, *p* = 0.090) or after the intervention (U = 291.00, *p* = 0.098) in reaction time. However, within-group analysis indicated a significant improvement in reaction time in the VRT + Medication group following the intervention (Z = -2.710, *p* = 0.007), with a large effect size (|r| ≈ 0.512), whereas the Medication-only group did not significantly change (Z = -1.526, *p* = 0.127). For the incongruent condition, between-group comparisons before the intervention revealed no significant difference (U = 329.50, *p* = 0.306), whereas a significant difference was observed after the intervention (U = 253.00, *p* = 0.035) in reaction time. Within-group analysis revealed a significant improvement in reaction time for the VRT + Medication group after the intervention (Z = -3.0512, *p* = 0.002), with a large effect size (|r| ≈ 0.577), whereas the medication-only group did not significantly change (Z = -0.843, *p* = 0.399).

For the congruent condition, no significant between-group differences were found before (U = 385.00, *p* = 0.899) or after the intervention (U = 387.00, *p* = 0.912) in accuracy. Within-group comparisons revealed a significant improvement in accuracy in the VRT + Medication group after the intervention (Z = -2.254, *p* = 0.024), whereas no significant change was observed in the Medication only group (Z = -1.715, *p* = 0.086). In the incongruent condition, between-group comparisons revealed no significant differences before (U = 281.00, *p* = 0.065) or after the intervention (U = 308.00, *p* = 0.142). However, within-group analysis indicated a significant improvement in accuracy in the VRT + Medication group after the intervention (Z = -4.032, *p* < 0.05), whereas no significant change was observed in the Medication-only group (Z = -0.418, *p* = 0.676).

### P300 response

P300 response latency and amplitude were recorded at the Fz, Cz, and Pz electrode sites before and after intervention in both groups. Before the intervention, P300 responses were identified in 9, 21, and 15 participants at Fz, Cz, and Pz, respectively, in the medication-only group and in 11, 20, and 23 participants at the same sites in the VRT + medication group. After the intervention, the number of participants with identifiable P300 responses increased in both groups: Fz (medication-only group: 10, VRT + medication group: 14), Cz (medication-only group: 23, VRT + medication group: 22), and Pz (medication-only group: 21, VRT + medication group: 26).

**Fig. 3:**
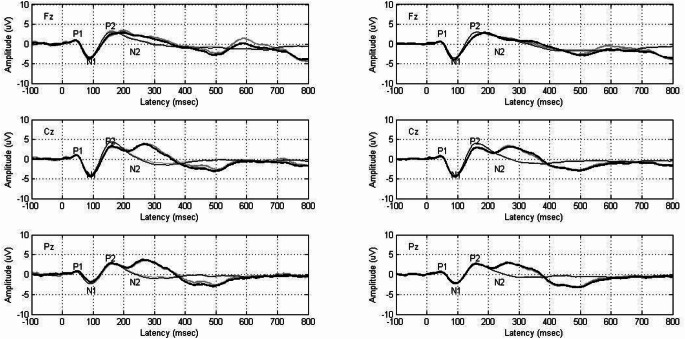
The grand average waveform of the P300 response recording of both groups recorded before and after intervention

Figure 3 shows grand-averaged waveforms for both groups before and after intervention across electrode sites. Light grey lines represent preintervention standard (dotted) and deviant (straight) waveforms, whereas black lines represent postintervention standard (dotted) and deviant (straight) waveforms.

The Shapiro‒Wilk test showed that P300 response latency and amplitude at Fz, Cz, and Pz were generally normally distributed, except for the amplitude at Fz in the VRT + Medication group after the intervention.

**Fig. 4:**
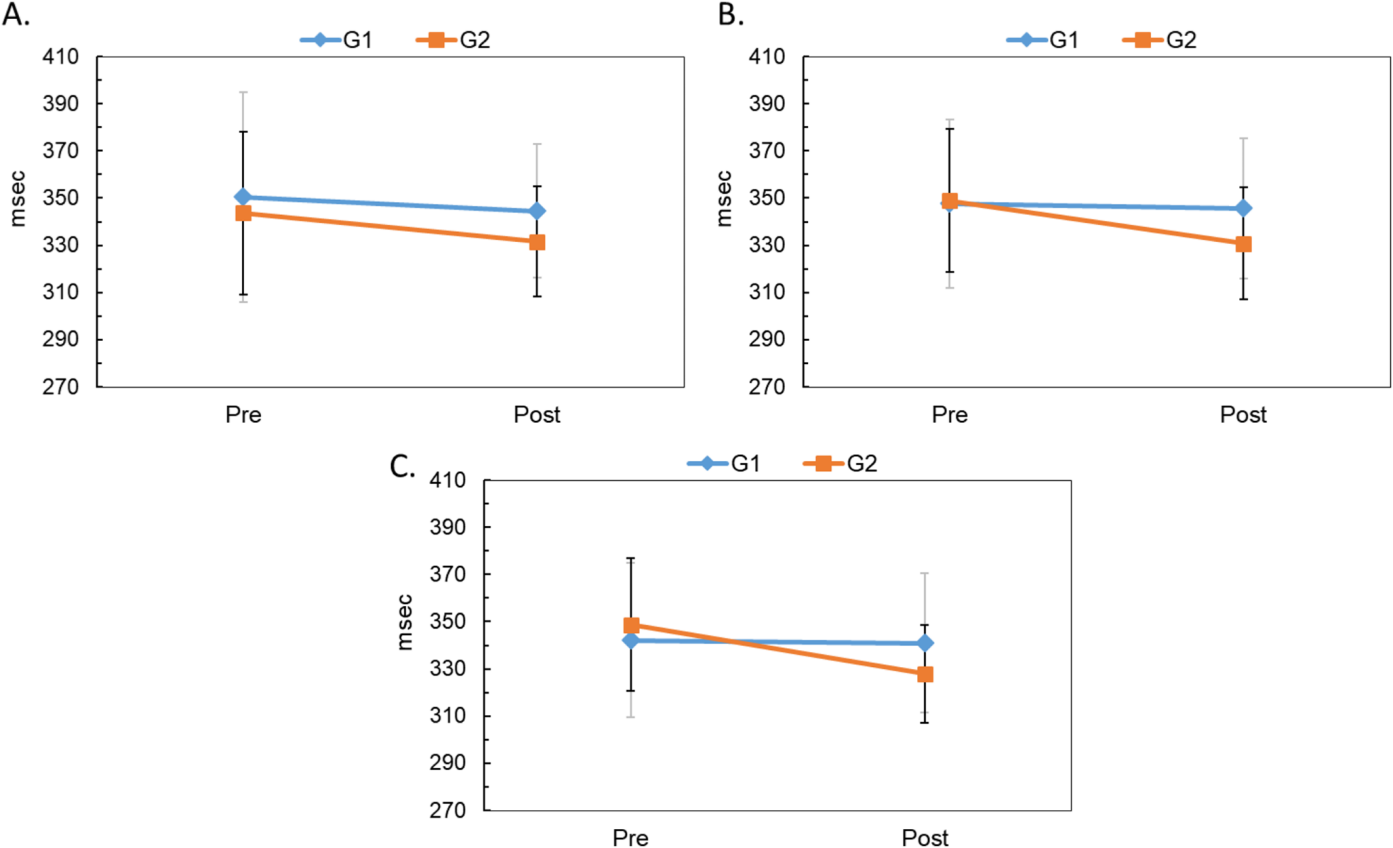
Mean and standard deviation (error bar) for P300 response latency recorded at Fz(panel **A**), Cz(panel **B**), and Pz(panel **C**) before and after intervention in both groups

Figure 4 illustrates the mean P300 response latency at Fz (panel A), Cz (panel B), and Pz (panel C) before and after intervention. After intervention, latency decreased across electrode sites in both groups, with a greater reduction in the VRT + Medication group. At Cz, repeated-measures ANOVA revealed a significant effect of intervention [F(1,34) = 16.45, *p* < 0.01] and a significant interaction between group and intervention [F(1,34) = 4.65, *p* = 0.038]. Paired t-test showed a significant reduction in latency for VRT + Medication group [t(17) = 4.100, *p* < 0.01] postintervention.

**Fig. 5:**
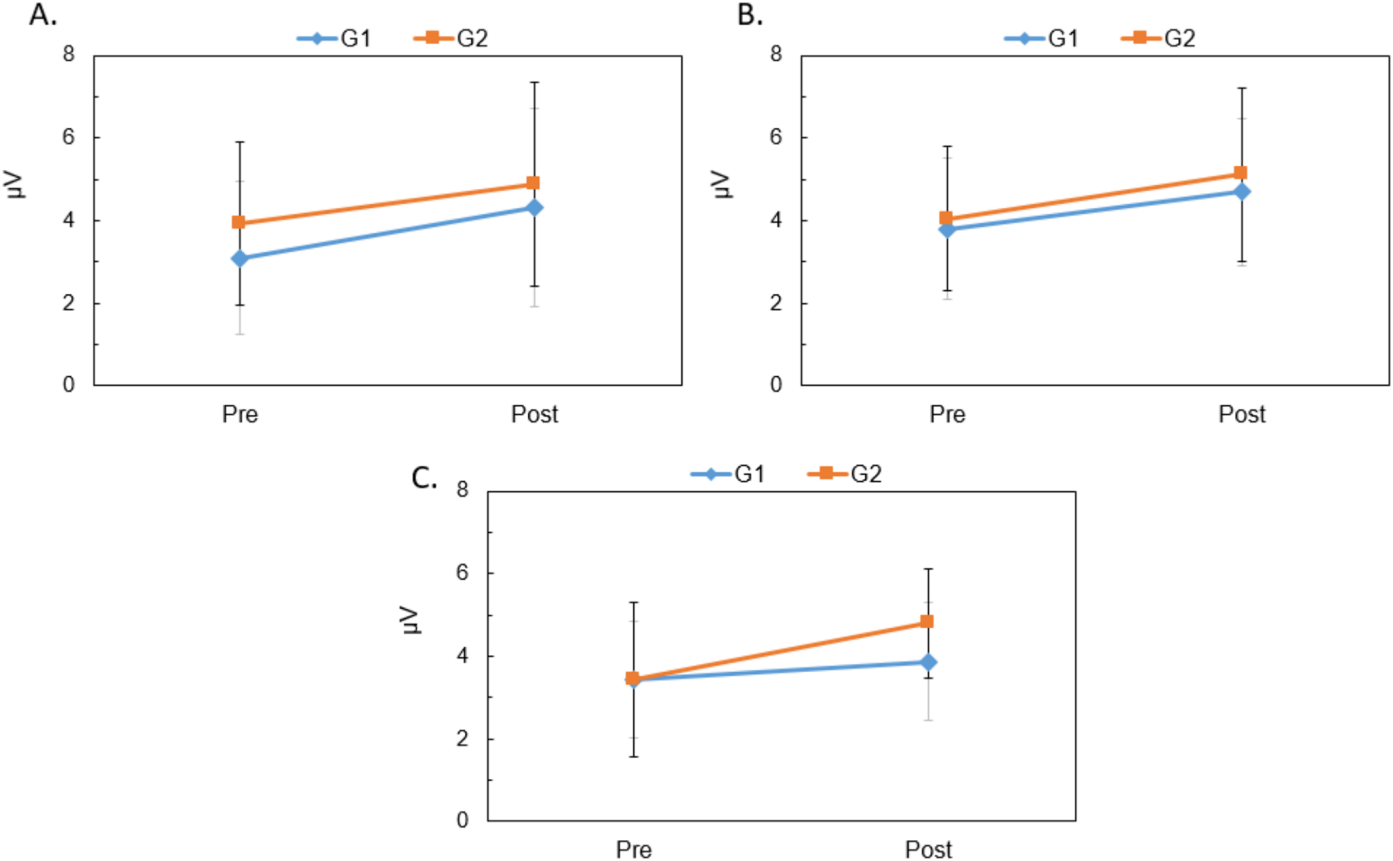
Mean and standard deviation (error bar) for P300 response amplitude recorded at Fz (panel **A**), Cz (panel **B**), and Pz (panel **C**) before and after intervention in both groups

Figure 5 shows the mean P300 response amplitude at Fz (panel A), Cz (panel B), and Pz (panel C). After the intervention, the amplitude increased in both groups, with a greater increase in the VRT + Medication group. Repeated-measures ANOVA at Cz revealed a significant effect of the intervention [F(1,34) = 12.48, *p* < 0.01], confirming improvement due to the intervention.

### Relationship between cognitive assessment and DHI

Pearson’s correlation analysis was used to evaluate the relationships between measures of cognitive function (P300 response latency, amplitude, digit span, task switching (reaction time and accuracy)) and DHI scores before and after the intervention in the two groups. Before intervention, the medication-only group showed a significant negative correlation between P300 response latency and DHI scores at Cz (*r* = -0.497, *p* = 0.022), whereas no significant correlations were found in the VRT + Medication group or at other electrode sites in either group. After the intervention, the medication-only group showed a significant positive correlation at Cz (*r* = 0.415, *p* = 0.049), but no significant correlations were found at other sites or in the VRT + Medication group. For the P300 response amplitude, there were no significant correlations with DHI scores in either group, either before or after the intervention. Similarly, no significant correlations were found between digit span and DHI scores or between task switching measures (reaction time and accuracy) and DHI scores in either the group before or after the intervention. In conclusion, the only significant correlations were observed between P300 response latency and DHI scores in the medication-only group at Cz, both before and after intervention.

## Discussion

The findings of the current study showed significant improvements in cognitive measures, particularly in the VRT + Medication group, where significant effects were observed in the digit span and task switching tests. Other studies have reported similar findings, with improvements in cognitive function after vestibular rehabilitation, although some cognitive measures, such as the Stroop test, showed only minor improvements [24]. In terms of P300 response, the present study revealed significant differences in the P300 response latency and amplitude at Cz in the VRT + Medication group, with the P300 response latency significantly changing after the intervention and between groups, whereas the amplitude improved postintervention without intergroup differences. These results align with previous studies that reported differences in P300 response latencies and amplitudes in patients with vestibular dysfunction [17, 18]. P300 response testing is valuable for assessing cognitive functions such as attention and memory in patients with vestibular-related dizziness or vertigo, as a lower amplitude and prolonged latency are linked to cognitive impairment [18].

The study also referenced the neuropsychological model based on Kahneman’s capacity model of attention, which suggests that individuals with vestibular dysfunction require more cognitive resources to maintain balance, reducing the resources available for other cognitive tasks [13]. The study administered the MMSE to ensure that no cognitive deficits affected the results. Vestibular rehabilitation significantly improved both subjective and objective cognitive measures, suggesting that the cognitive abilities affected by vestibular dysfunction can be restored through vestibular rehabilitation. The participants also reported reduced symptoms and improved quality of life after therapy. Furthermore, other studies have shown that combining vestibular rehabilitation with cognitive behavioral therapy (CBT) can reduce symptoms of vertigo and improve cognitive function [21, 25].

With respect to the relationship between the cognitive assessment score and DHI score, the current study revealed a significant negative correlation between the P300 response latency at Cz and DHI in the medication-only group before the intervention. After the intervention, the medication-only group showed a positive correlation, whereas no significant correlations were found in the VRT + medication group. No significant correlation was detected between the P300 response amplitude and DHI in either group. Other studies have also explored correlations between cognitive and vestibular assessments, but findings on the relationship between P300 response and DHI are limited. Some studies reported moderate correlations between vestibular function and cognitive performance [6], whereas others reported correlations between DHI and emotional or functional domains of cognitive failure [26, 27]. The DHI assesses the impact of vestibular dysfunction on quality of life, whereas the P300 response is an objective cognitive measure, which explains the lack of consistent findings between these two measures.

While the current effort provides novel insights into the benefits of VRT on quality of life and certain aspects of cognitive function, there are limitations that suggest future research directions. The relatively young mean age of participants may limit generalizability to older adults, where age-related cognitive decline could amplify vestibular-cognitive interactions; future studies should explore these effects in cohorts aged ≥ 65 years. Although the current study design isolates VRT’s contribution by comparing medication-only versus VRT + medication groups, a standalone VRT-only group could have provided more insight into VRT’s independent impact. The broad classification of participants as having chronic dizziness or vertigo without pathological specificity may obscure potential differences across underlying causes, despite aligning with our symptomatic focus. This highlights opportunities for further research in specific dizziness or vertigo groups, such as peripheral vestibular pathologies or central vestibular disorders.

## Conclusion

The findings of this study highlight the positive effects of vestibular rehabilitation therapy on cognitive function and quality of life in individuals with chronic dizziness or vertigo. Significant improvements were observed in cognitive measures, particularly in the digit span and task switching tests, with increased P300 response latency and amplitude in the VRT + Medication group. These results support the hypothesis that VRT not only alleviates physical symptoms but also contributes to cognitive improvements. Additionally, while the relationship between cognitive function and quality of life, as measured by the DHI, showed mixed correlations, the overall impact of VRT suggests its potential as a beneficial therapeutic approach for individuals affected by vestibular dysfunction.
